# The Polyamine Spermidine Modulates the Production of the Bacterial Genotoxin Colibactin

**DOI:** 10.1128/mSphere.00414-19

**Published:** 2019-10-02

**Authors:** Camille V. Chagneau, Christophe Garcie, Nadège Bossuet-Greif, Sophie Tronnet, Alexander O. Brachmann, Jörn Piel, Jean-Philippe Nougayrède, Patricia Martin, Eric Oswald

**Affiliations:** a CHU Toulouse, Service de Bactériologie-Hygiène, Toulouse, France; b IRSD, Université de Toulouse, INSERM, INRA, ENVT, UPS, Toulouse, France; c Institute of Microbiology, Eidgenössische Technische Hochschule (ETH), Zurich, Switzerland; University of Kentucky

**Keywords:** *Escherichia coli*, biosynthesis, colorectal cancer, genotoxic colibactin, polyamines

## Abstract

Colibactin is a polyketide/nonribosomal peptide produced by Escherichia coli strains that harbor the *pks* island. This toxin induces DNA double-strand breaks and DNA interstrand cross-links in infected eukaryotic cells. Colibactin-producing strains are found associated with colorectal cancer biopsy specimens and promote intestinal tumor progression in various murine models. Polyamines are small polycationic molecules produced by both microorganisms and eukaryotic cells. Their levels are increased in malignancies, where they contribute to disease progression and metastasis. In this study, we demonstrated that the endogenous spermidine synthase SpeE is required for full genotoxic activity of colibactin-producing E. coli. Supplying spermidine in a Δ*speE pks*^+^ E. coli strain restored genotoxic activity. Spermidine is involved in the autotoxicity linked to colibactin and is required for direct damaging activity on DNA. The production of the colibactin prodrug motif is impaired in Δ*speE* mutants. Therefore, we demonstrated that spermidine has a direct impact on colibactin synthesis.

**IMPORTANCE** Colibactin-producing Escherichia coli strains are associated with cancerous and precancerous colorectal tissues and are suspected of promoting colorectal carcinogenesis. In this study, we describe a new interplay between the synthesis of the genotoxin colibactin and the polyamine spermidine. Polyamines are highly abundant in cancer tissue and are associated with cell proliferation. The need for spermidine in genotoxic activity provides a new perspective on the role of these metabolites in the pathogenicity of colibactin-producing E. coli strains in colorectal cancer.

## Abstract (precis)

Colibactin-producing Escherichia coli strains are associated with cancerous and precancerous colorectal tissues and are suspected of promoting colorectal carcinogenesis. In this study, we describe a new interplay between the synthesis of the genotoxin colibactin and the polyamine spermidine. Polyamines are highly abundant in cancer tissue and are associated with cell proliferation. The need for spermidine in genotoxic activity provides a new perspective on the role of these metabolites in the pathogenicity of colibactin-producing E. coli strains in colorectal cancer.

## INTRODUCTION

The genotoxin colibactin is a secondary microbial metabolite synthetized by Escherichia coli and other enterobacteria. The genetic determinant of colibactin is a 54-kb gene cluster, the *pks* genomic island (1). This highly conserved pathogenicity island is predominately found in E. coli strains of the phylogenetic group B2 and in some other species of *Enterobacteriaceae* (1, 2). The *pks* island carries the genes *clbA* to *clbS*, which encode modular nonribosomal peptide synthetases (NRPSs), polyketide synthases (PKSs), and accessory enzymes (1). This complex biosynthetic assembly line is responsible for the synthesis of colibactin, which belongs to the chemical family of hybrid polyketide/nonribosomal peptide (PK-NRP) compounds. Mature colibactin has a complex structure of highly unstable and reactive molecules forming DNA adducts (3–12). However, the complete structure of genotoxic colibactin is not yet fully elucidated.

Colibactin is genotoxic for eukaryotic (1) as well as prokaryotic cells when the ClbS resistance protein is not produced (13). Colibactin-induced DNA damages result from the formation of interstrand DNA cross-links leading to DNA double-strand breaks (12, 14). These DNA damages can lead to gene mutations, chromosomal instability, and senescence (15, 16), and in various mouse models colibactin-producing E. coli strains promote intestinal tumor progression (17–20).

Epidemiological studies show a high prevalence of *pks*-carrying E. coli strains in biopsy specimens from colorectal cancer patients (17, 21, 22). However, the presence of a *pks*^+^ strain in gut microbiota is not sufficient to induce colorectal cancer. Other factors have been shown to promote cell transformation and/or to potentiate the bacterial genotoxicity of these bacteria. For example, deoxynivalenol, a food contaminant, exacerbates the genotoxic effect linked to colibactin in animals colonized by *pks*^+^ E. coli (23). Environmental factors such as iron concentration can also directly regulate colibactin production (24). However, inflammation is a more significant factor, which seems to be required in colibactin-associated carcinogenesis (17, 20). Inflammation itself is critical for tumor progression (by promoting cell proliferation, survival, and migration) but it also drives modifications in microbiota composition and the expansion of E. coli (25). It has been shown that a proinflammatory cancer microenvironment could increase the expression of *clb* genes, at least at a transcriptional level (26). Recently, an association between colibactin-producing E. coli and enterotoxigenic Bacteroides fragilis, another procarcinogenic bacterial species, was also noted on the colonic mucosa of patients with familial adenomatous polyposis (FAP), who are highly susceptible to colorectal cancer (20). A synergy between these two bacterial species was observed in tumor formation in an FAP murine model (20).

Polyamines are essential for cell proliferation and have been shown to play a crucial role in carcinogenesis (27, 28). They are small aliphatic molecules involved in protein synthesis and regulation, DNA integrity, stress resistance. (29). Infections by microorganisms and chronic inflammation can interfere with polyamine catabolism and increase the formation of damaging oxidative compounds, contributing *in fine* to carcinogenesis (30, 31). Polyamines are found at high concentrations in colorectal cancer tissues even at precancerous states (32) but are also produced by intestinal bacteria such as E. coli (33).

In this study, we investigated the putative roles of polyamines in colibactin genotoxic activity. We demonstrate that spermidine is involved in colibactin synthesis and the associated genotoxicity.

## RESULTS

### The spermidine biosynthetic pathway is required for full genotoxicity of colibactin-producing E. coli.

**FIG 1:**
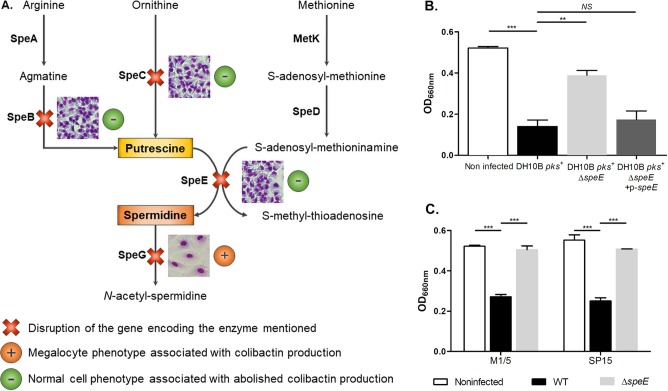
Endogenous spermidine biosynthesis is involved in the E. coli colibactin-associated megalocytosis phenotype. (A) Presentation of the polyamine biosynthetic pathways (34). DH10B *pks*^+^ mutants with mutations of genes *speB*, *speC*, *speE*, and *speG* were tested for the megalocytosis phenotype in infected HeLa cells, as previously described (1). The phenotypes of HeLa cells resulting from infection with the different mutants are shown. (B and C) Cytotoxic effects of colibactin produced by E. coli strains DH10B *pks*^+^, M1/5, and SP15 and their derivatives were determined by quantification of megalocytosis. At the end of HeLa cell infection, the methylene blue protein staining was quantified by measurement of absorbance at the optical density at 660 nm. The multiplicity of infection (MOI) was 200. Data were pooled from three independent experiments. ***, *P < *0.001, and **, *P < *0.01, by 1-way analysis of variance (ANOVA). All bar graphs show mean values ± standard errors of the mean (SEM). NS, not significant.

**FIG 2:**
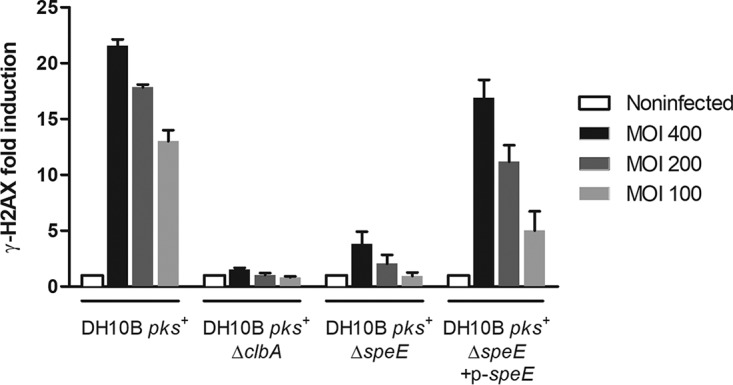
Deletion of the spermidine synthase SpeE impacts full genotoxicity of colibactin-producing E. coli. The production of colibactin by E. coli strain DH10B *pks*^+^ and derivatives was determined by quantification of H2AX phosphorylation, which correlates with DNA damage resulting from the genotoxic effect of colibactin. E. coli strain DH10B *pks*^+^, the Δ*clbA* mutant (negative control), and the Δ*speE* mutant and its complemented derivative were cocultivated with HeLa cells in an In-Cell Western assay as previously described (35). The multiplicity of infection (MOI [i.e., the number of bacteria per cell) ranged from 100 to 400. Data shown in the graph are representative of three independent experiments. All bar graphs show mean values ± SEM.

To test the impact of the endogenous spermidine-putrescine pathway on colibactin-producing E. coli genotoxic activity, mutants inactivated for the *speB*, *speC*, *speE*, and *speG* genes (Fig. 1A) were engineered in E. coli strain DH10B, which harbors the *pks* island on a bacterial artificial chromosome (Table 1) (34). The production of colibactin by each mutant was first monitored through bacterium-host cell interactions and subsequent observation of the formation of large senescent cells (megalocytosis) (Fig. 1). Inactivation of *speB*, *speC*, and *speE* genes but not of *speG* resulted in a decrease in the megalocytosis effect (Fig. 1A), with no effect on bacterial growth (data not shown). Therefore, spermidine biosynthesis, and especially the final step catalyzed by the spermidine synthase SpeE, has more impact on colibactin genotoxic activity than putrescine biosynthesis. We confirmed the loss of the megalocytosis phenotype in the Δ*speE* mutant and its restoration after plasmid complementation (Fig. 1B). The inactivation of *speE* in *pks*^+^ E. coli from other genetic backgrounds (i.e., the E. coli M1/5 commensal strain and the E. coli SP15 pathogenic strain isolated from neonatal meningitis) also resulted in a decrease in the megalocytosis effect (Table 1 and Fig. 1C). We then measured the genotoxic activity of the bacteria by quantifying histone H2AX phosphorylation in response to DNA damage in the infected cells (1, 15, 35). We confirmed that the inactivation of the *speE* gene in the DH10B *pks*^+^ strain markedly decreased the genotoxic effect induced by colibactin (Fig. 2), whereas the complemented mutant was fully genotoxic (Fig. 2).

**TABLE 1:** E. coli strains and plasmids used in this study

Strain or plasmid	Genotype or phenotype	Source
E. coli strains		
DH10B *pks*^+^	K-12 laboratory strain carrying pBAC*pks*, Cm^r^	1
DH10B *pks*^+^ Δ*speB*	*speB* mutant of strain DH10B pBAC*pks*, Cm^r^ Kan^r^	This study
DH10B *pks*^+^ Δ*speC*	*speC* mutant of strain DH10B pBAC*pks*, Cm^r^ Kan^r^	This study
DH10B *pks*^+^ Δ*speE*	*speE* mutant of strain DH10B pBAC*pks*, Cm^r^ Kan^r^	This study
DH10B *pks*^+^ Δ*speG*	*speG* mutant of strain DH10B pBAC*pks*, Cm^r^ Kan^r^	This study
DH10B *pks*^+^ Δ*speE* + p-*speE*	DH10B *pks^+^* Δ*speE* carrying p-*speE*, Cm^r^ Kan^r^ Amp^r^	This study
DH10B *pks*^+^ Δ*clbA*	*clbA* mutant of strain DH10B pBAC*pks*, Cm^r^	1
DH10B *pks*^+^ Δ*clbS*	*clbS* mutant of strain DH10B pBAC*pks*, Cm^r^	13
DH10B *pks*^+^ Δ*speE* Δ*clbS*	*speE clbS* double mutant of strain DH10B pBAC*pks*, Cm^r^	This study
M1/5	Commensal E. coli strain isolated from feces of a healthy adult, B2 phylogenetic group, colibactin genotoxin producer	35
M1/5 Δ*speE*	*speE* mutant of strain M1/5, Kan^r^	This study
SP15	Extraintestinal pathogenic *E. coli* strain isolated from spinal fluid of neonate with meningitis, O18:K1 serotype, colibactin genotoxin producer	50
SP15 Δ*speE*	*speE* mutant of strain SP15, Kan^r^	This study
SP15 Δ*speG*	*speG* mutant of strain SP15, Kan^r^	This study

Plasmid		
p-*speE*	pSC-A plasmid carrying wild-type *speE* gene, Amp^r^ Kan^r^	This study

### Spermidine is required for full genotoxicity of colibactin-producing E. coli.

**FIG 3:**
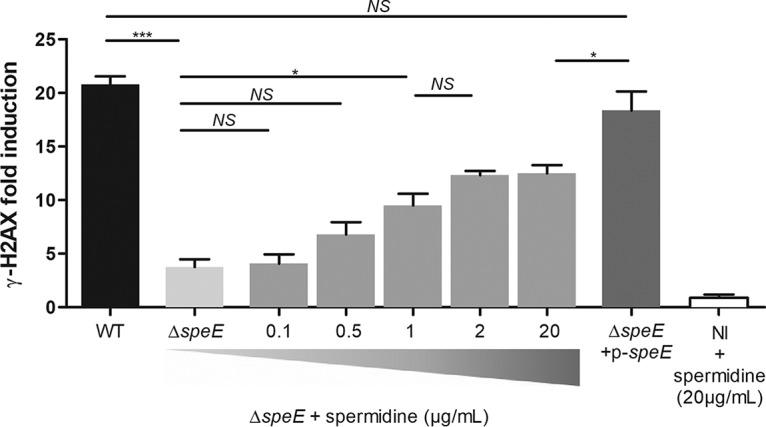
Exogenous spermidine restores colibactin-associated genotoxicity in a Δ*speE* mutant. Colibactin-mediated genotoxicity was determined by infection of HeLa cells with E. coli strain DH10B *pks*^+^ and derivatives and quantification of H2AX phosphorylation. When indicated, spermidine (μg/ml) was added to the interaction medium during infection. MOI = 400. NI, noninfected. Data were combined from three independent experiments. ***, *P <* 0.001, and *, *P <* 0.05, by 1-way ANOVA. All bar graphs show mean values ± SEM. NS, not significant.

In order to determine whether the decreased genotoxicity of the Δ*speE* mutant is associated with the spermidine synthase SpeE or to spermidine itself, we added increasing concentrations of spermidine in the interaction medium during HeLa cell infection (Fig. 3). The production of colibactin was again measured by quantifying histone H2AX phosphorylation (Fig. 3) and megalocytosis assay (see Fig. S1 in the supplemental material). Spermidine supplementation of the Δ*speE* mutant restored its genotoxicity in a dose-dependent manner (Fig. 3).

10.1128/mSphere.00414-19.4 FIG S1 Spermidine restores megalocytosis after infection with a Δ*speE* mutant. Cytotoxic effects of colibactin produced by E. coli strain SP15 and its derivative were determined by quantification of megalocytosis. When indicated, spermidine was added at 20 μg/ml. At the end of HeLa cell infection, the methylene blue protein staining was quantified by measuring absorption at an optical density of 660 nm. The multiplicity of infection (MOI) was 200. Data were pooled from three independent experiments. ***, *P* < 0.001, **, *P* < 0.01, and *, *P* < 0.05, by 1-way analysis of variance (ANOVA). All bar graphs show mean values ± standard errors of the mean (SEM). ns, not significant. Download FIG S1, TIF file, 0.7 MB. Copyright © 2019 Chagneau et al. 2019 Chagneau et al. This content is distributed under the terms of the Creative Commons Attribution 4.0 International license.

**FIG 4:**
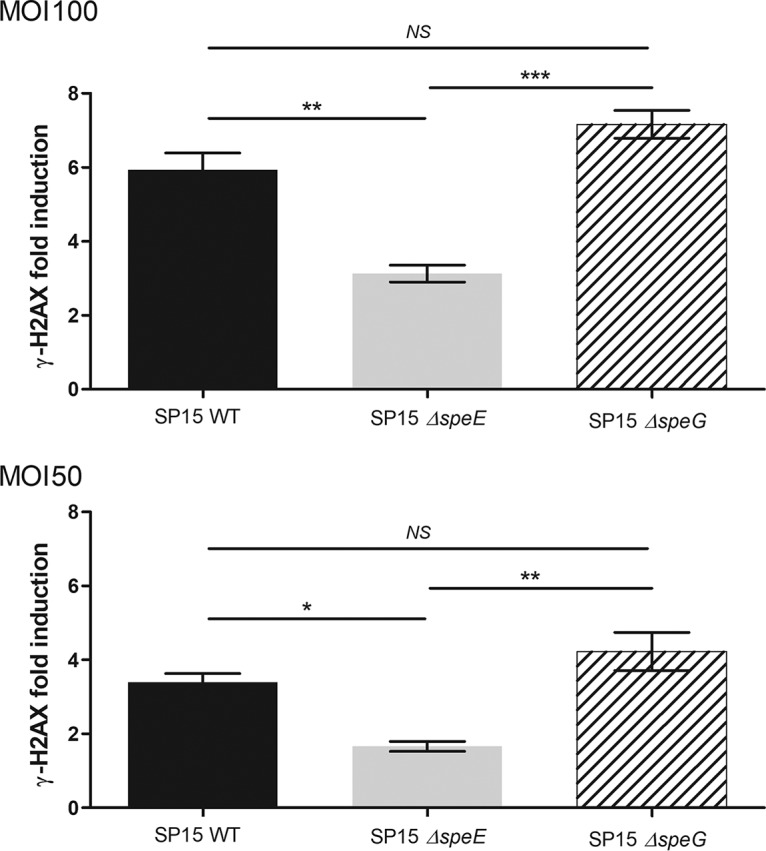
Mutation of the spermidine acetyltransferase SpeG leads to an increase in colibactin-associated genotoxicity. Colibactin-mediated genotoxicity was determined by infection of HeLa cells with E. coli strain SP15 and Δ*speE* and Δ*speG* mutants and quantification of H2AX phosphorylation. MOI = 50 and 100. Data were pooled from two independent experiments in duplicate. ***, *P <* 0.001, **, *P <* 0.01, and *, *P <* 0.05, by 1-way ANOVA. All bar graphs show mean values ± SEM. NS, not significant.

The spermidine acetyltransferase SpeG catalyzes spermidine acetylation into physiologically inert *N*-acetylspermidine. While screening the impact of the putrescine-spermidine pathway on colibactin-associated megalocytosis phenotype, we observed no Δ*speG* mutant defect (Fig. 1A). We hypothesized that by abolishing SpeG activity, spermidine would accumulate in the bacteria and boost the genotoxic activity of *pks*^+^ E. coli. To test this hypothesis, we quantified H2AX phosphorylation in response to DNA damages after HeLa cell infection by E. coli SP15 and deletion mutants of *speE* and *speG* genes (Fig. 4). Compared to the wild-type strain, we observed a decrease in the genotoxic activity of SP15 Δ*speE* (Fig. 4). However, SP15 Δ*speG* with impaired catabolism of spermidine induced 20% more DNA damage than the wild-type SP15 strain (Fig. 4). These results confirmed that spermidine is the key player in the interaction between the putrescine-spermidine pathway and colibactin-associated genotoxic activity. However, a Δ*potD* spermidine import mutant of E. coli strain SP15 was not impaired in its genotoxicity (see Fig. S2 in the supplemental material), suggesting that spermidine endogenous production is sufficient to support colibactin production in this *in vitro* assay. Furthermore, we tested other polyamines and showed that spermine and norspermidine could restore the genotoxic activity in a Δ*speE* mutant (see Fig. S3 in the supplemental material).

10.1128/mSphere.00414-19.5 FIG S2 A spermidine import mutant is not impaired in its colibactin-related genotoxicity. Colibactin-mediated genotoxicity was determined by infection of HeLa cells with E. coli strain SP15 and Δ*speE*, Δ*potD*, and Δ*speE* Δ*potD* mutants and quantification of H2AX phosphorylation. MOI = 100. Data were pooled from two independent experiments in duplicate. ***, *P* < 0.001 by 1-way ANOVA. All bar graphs show mean values ± SEM. NS, not significant. Download FIG S2, TIF file, 1.4 MB. Copyright © 2019 Chagneau et al. 2019 Chagneau et al. This content is distributed under the terms of the Creative Commons Attribution 4.0 International license.

10.1128/mSphere.00414-19.6 FIG S3 Various polyamines can restore colibactin-associated genotoxicity in an SP15 Δ*speE* mutant. Colibactin-mediated genotoxicity was determined by infection of HeLa cells and quantification of H2AX phosphorylation for E. coli strain SP15 and the Δ*speE* mutant. When indicated, polyamines (3.44 μM) were added to the interaction medium during infection. MOI = 100. Data are pooled from three independent experiments. ***, *P* < 0.001, and **, *P* < 0.01, by 1-way ANOVA. All bar graphs show mean values ± SEM. Download FIG S3, TIF file, 0.7 MB. Copyright © 2019 Chagneau et al. 2019 Chagneau et al. This content is distributed under the terms of the Creative Commons Attribution 4.0 International license.

### Spermidine is directly involved in colibactin biosynthesis.

**FIG 5:**
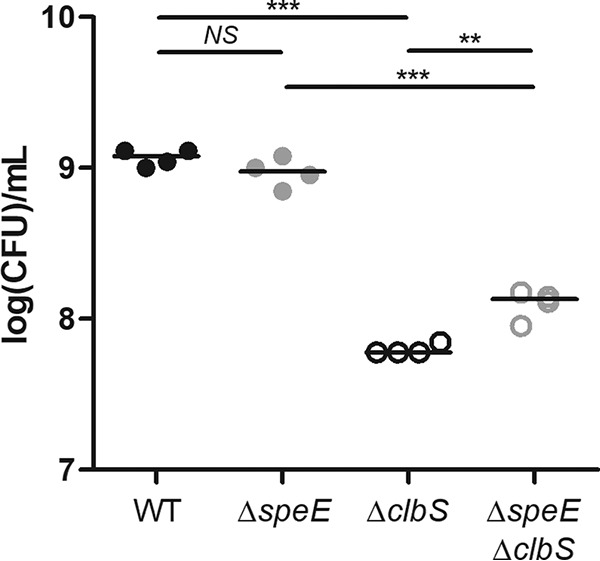
Deletion of the spermidine synthase SpeE decreases colibactin autotoxicity in a Δ*clbS* mutant. Enumeration of culturable bacterial cells in the stationary growth phase. The bacteria were pregrown in LB to reach an exponential growth (OD_600_ = 0.4). A total of 2 × 10^6^ bacteria/ml were then inoculated in LB and grown for 17 h before being plated on LB agar plates to determine CFU. The median and individual results of four independent experiments are shown. ***, *P <* 0.001, and **, *P < *0.01, by 1-way ANOVA. NS, not significant.

To determine the level at which spermidine acts, we quantified the autotoxicity linked to colibactin production directly in bacteria. In fact, DNA damage can occur in toxigenic bacteria mutated for the ClbS resistance protein, leading to activation of the SOS response and then decreased growth (13). We constructed a Δ*speE* Δ*clbS* double mutant in the E. coli strain DH10B *pks*^+^ (Table 1) and compared its growth after 17 h in LB broth to Δ*clbS* and Δ*speE* mutants. As expected, there was a decrease in the number of Δ*clbS* mutant CFU compared to the wild-type strain (Fig. 5). Reduced CFU counts were also observed for the Δ*speE* Δ*clbS* double mutant compared to the Δ*speE* mutant, but to a lesser extent than for the Δ*clbS* mutant (Fig. 5). Therefore, *speE* mutation significantly decreases colibactin autotoxicity in a Δ*clbS* mutant, suggesting decreased production of genotoxin by the bacteria when the polyamine pathway is disrupted (Fig. 5).

**FIG 6:**
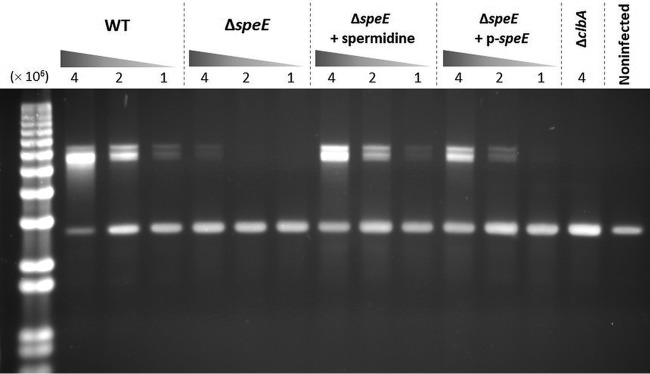
Spermidine is involved in DNA cross-linking activity. DNA cross-linking was observed by cultivating the E. coli strains with linearized plasmid DNA. Two hundred nanograms of linearized pUC19 plasmid was added to 1 × 10^6^, 2 × 10^6^, or 4 × 10^6^ bacteria/well of wild-type (WT) E. coli strain DH10B *pks*^+^, the Δ*clbA* and Δ*speE* mutants, and the complemented derivative. When indicated, 20 μg/ml of spermidine was added to the interaction medium during infection. DNA was then purified, loaded on agarose gel, and run under alkaline denaturing conditions. DNA with covalent interstrand cross-links is nondenaturable and displays delayed migration compared to denatured single-stranded DNA (lower band). This image is representative of three independent experiments.

To confirm the involvement of spermidine in colibactin production, we monitored the DNA cross-linking activity of colibactin-producing E. coli in exogenous DNA, in an acellular assay. Following incubation with bacteria, plasmid DNA was purified and analyzed by electrophoresis on agarose gel under denaturing conditions to highlight DNA interstrand cross-linking with delayed migration compared to linearized single-stranded DNA (Fig. 6) (14). This experiment showed that *speE* mutation greatly attenuates DNA cross-linking activity, which was observed only for the highest bacterial dose (Fig. 6). The ability of the Δ*speE* mutant to induce DNA cross-links was restored by either exogenous addition of spermidine or *speE* complementation (Fig. 6) or transcomplementation by polyamine production of a wild-type E. coli strain without *pks* island in coculture (see Fig. S4 in the supplemental material). These experiments performed without eukaryotic host cells suggest that spermidine has a direct role in the production of colibactin, which results in fully genotoxic bacteria.

10.1128/mSphere.00414-19.7 FIG S4 Transcomplementation of genotoxic activity of Δ*speE* mutant by a wild-type E. coli strain. DNA cross-linking was observed by cultivating the E. coli strains with linearized plasmid DNA. Four hundred nanograms of linearized pUC19 plasmid was added to 6 × 10^6^ bacteria/well of the E. coli DH10B *pks*^+^ Δ*speE* mutant, DH10B/pBAC (wild-type [wt] E. coli), and cocultures (Δ*speE* + wt E. coli) of these strains, either since the 2-h preculture (preculture) or only since the interaction with DNA (interaction). When indicated, 2 μg/ml of spermidine was added to the interaction medium during infection. DNA was then processed as in Fig. 6. This image represents two independent experiments. Download FIG S4, EPS file, 0.9 MB. Copyright © 2019 Chagneau et al. 2019 Chagneau et al. This content is distributed under the terms of the Creative Commons Attribution 4.0 International license.

**FIG 7:**
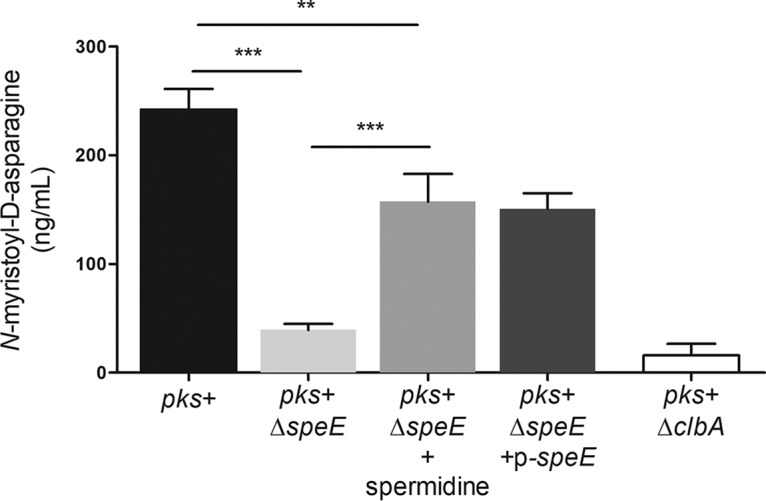
Spermidine is involved in colibactin biosynthesis. The colibactin prodrug motif *N*-myristoyl-d-asparagine, produced by the wild-type (WT) E. coli strain DH10B *pks*^+^, the Δ*clbA* and Δ*speE* mutants, and the complemented derivative, was quantified by liquid chromatography-mass spectrometry (LC-MS). Bacteria were cultivated at 37°C for 18h in DMEM-HEPES, supplemented when indicated with 20 μg/ml of spermidine, and the levels of bacterial growth were similar under all conditions. The data presented in the graph were obtained from four biological replicates. ***, *P < *0.001, and **, *P < *0.01, by 1-way ANOVA. All bar graphs show mean values ± SEM.

Considering that mature colibactin is not yet directly quantifiable, we took advantage of the stability of the *N*-myristoyl-d-asparagine moiety cleaved by the ClbP peptidase in the late activation step of inactive precolibactin (3, 4) to indirectly access the amount of colibactin produced, using LC-MS (36) (Fig. 7). Quantification of *N*-myristoyl-d-asparagine in culture supernatants revealed that the amount of colibactin prodrug motif was drastically decreased in the Δ*speE* mutant compared to the E. coli DH10B *pks*^+^ strain, and partially restored by complementation with p-*speE* (Fig. 7). Supplementation of spermidine during growth increased the production of *N*-myristoyl-d-asparagine by the Δ*speE* mutant (Fig. 7).

We tested whether spermidine altered *clb* gene expression by using *clb* reporter strains previously designed in E. coli Nissle 1917 (37) (see Fig. S5 and Text S1 in the supplemental material). We observed modifications of expression after both Δ*speE* mutation and spermidine supplementation, suggesting that spermidine can modify expression of *pks* genes.

10.1128/mSphere.00414-19.1 TEXT S1 Supplemental material and methods with reference. Download Text S1, DOCX file, 0.02 MB. Copyright © 2019 Chagneau et al. 2019 Chagneau et al. This content is distributed under the terms of the Creative Commons Attribution 4.0 International license.

10.1128/mSphere.00414-19.8 FIG S5 Spermidine modulates the expression of genes of the *pks* island. Shown are the growth kinetics (OD_600_ [dotted lines]) and relative OD_600_-standardized relative luminescence units (RLU/OD_600_ [solid lines]) of the WT and Δ*speE* mutant E. coli Nissle 1917 (EcN) *clbQ-lux* (A), *clbR-lux* (B), *clbA-lux* (C) and *clbB-lux* (D) fusion strains grown at 37°C in DMEM-HEPES supplemented with 20 μg/ml spermidine or unsupplemented. The data shown were obtained from three biological replicates and are pooled from three independent experiments. All bar graphs show mean values ± SEM. Download FIG S5, TIF file, 1.1 MB. Copyright © 2019 Chagneau et al. 2019 Chagneau et al. This content is distributed under the terms of the Creative Commons Attribution 4.0 International license.

## DISCUSSION

Our work demonstrates the role of spermidine in the production of the genotoxin colibactin. We demonstrate that the endogenous spermidine biosynthesis pathway is involved in colibactin-associated genotoxicity. As a Δ*speE* mutant is impaired in its genotoxic activity in acellular DNA cross-linking assay, a mechanism mediated by the targeted eukaryotic cell (such as invasion) was ruled out. Spermidine is not necessary, however, as traces of colibactin-related toxicity remain after *speE* mutation, as demonstrated both by autotoxicity in the Δ*speE* Δ*clbS* double mutant and DNA cross-link formation at the highest bacterial concentration. Furthermore, *N*-myristoyl-d-asparagine, a metabolite directly linked to colibactin synthesis, also undergoes quantitative alteration when the spermidine pathway is inactivated, showing a direct involvement of spermidine in production of the toxin. Not only exogenous spermidine but also other polyamines such as spermine and norspermidine can restore colibactin biosynthesis in Δ*speE* mutants. Noteworthy, the three polyamines that cause complementation share structural characteristics: i.e., 3 or more amine functions, 2 of which are free at the ends. Proposed colibactin and precolibactin structures do not contain any spermidine or spermidine-derived motif (3–12). Thus, polyamines may likely be involved in colibactin synthesis with regard to monitoring its regulation. Precisely we have observed changes in the expression of different *clb* genes related to *speE* mutation or spermidine supplementation. Although the precise molecular mechanism remains elusive, polyamines are known to be major regulators in E. coli gene expression, especially at the translation step (38). Indeed, enhancement of mRNA and ribosome interaction leads to increased translation of proteins that belong to the “polyamine modulon” as it is called by the Igarashi and Kashiwagi team (38). Alternatively, polyamines could also act through facilitating transport or export of the genotoxin.

Spermidine and polyamines have been described as important players in different host-pathogen relationships. In the plague agent Yersinia pestis, for instance, an intact putrescine-spermidine pathway is essential to achieve biofilm formation, which is in turn important for transmission by fleas (39). A higher virulence linked to an increase in the spermidine content exists in *Shigella* sp., a bacterium responsible for dysentery and closely related to E. coli (40). During the pathoadaptive evolutionary changes in E. coli that led to this pathogen, the *speG* gene became defective due to diverse mutations. Spermidine accumulation in bacteria is a key factor for the invasion of macrophages in the pathophysiology of *Shigella* sp. and entero-invasive E. coli (EIEC), which share the same invasive process (40, 41). The production and response of colicin E7, an E. coli bacteriocin, are also regulated by polyamines (42). Spermidine and putrescine are indeed important for triggering toxin production after DNA damage because polyamines are mediators of the SOS response that regulates colicin E7. Exogenous spermidine decreases the susceptibility of E. coli to colicin E7, which can be attributed to a downregulation of the transporters of the toxin by the polyamine (42).

Spermidine is highly linked to cancer, especially colorectal cancer. Polyamines are associated with cell proliferation. Therefore, host cell polyamine content is upregulated in colorectal cancer tissues. It relies on both an increase in polyamine production and impaired catabolism (28). The host is not the only source of polyamine in the digestive tract. Bacteria in gut microbiota also produce polyamines and are one of the main sources of these compounds in the lower intestine (43). The diversity of the microorganisms makes the metabolism and composition of the polyamine pool diverse (44, 45), and it can be even more complex as a result of collective pathways within microorganisms as well as polyamines produced by eukaryotic cells (46). Food is another source of polyamines in the gut, especially nutrients such as fruits, wheat germs, cheeses, mushrooms, and nuts. (47). In our study, we demonstrated that exogenous spermidine produced by another E. coli strain could support colibactin biosynthesis by a spermidine mutant. This strongly suggests that in the gut, where spermidine is abundant, colibactin-producing bacteria can take advantage of this metabolite, whatever the origin of its production (microbiota, host, or food). Recently, the external supply of polyamine spermidine was also suggested to extend life span and to provide neuroprotective and cardioprotective properties, for example (48). To date, the literature on these beneficial effects of spermidine has been limited to model organisms and to epidemiological studies that link dietary uptake with longevity or pathology. Clinical trials aimed at increasing the uptake of this polyamine or even the administration of probiotics that enhance the microbial synthesis appear feasible (48). However, whether spermidine supplementation in humans would increase tumor growth or modify microbiota remains unknown and should be addressed in light of the fact that a polyamine-enriched environment could promote and may even trigger the production of the genotoxin colibactin. This reinforces the hypothesis of deleterious synergy between microbiota and host cell metabolism during carcinogenesis.

## MATERIALS AND METHODS

### Bacterial strains, mutagenesis, and growth conditions.

The bacterial strains used in this study are listed in Table 1 and Table S1 in the supplemental material (Text S1). For genetic manipulations, E. coli strains were routinely grown at 37°C under shaking in 5 ml of lysogeny broth (LB Lennox; Invitrogen). Appropriate antibiotics were added to the medium when required (chloramphenicol, 25 μg/ml; kanamycin, 50 μg/ml; carbenicillin, 50 μg/ml). Inactivation of the genes *speB*, *speC*, *speE*, and *speG* was performed by using the lambda Red recombinase method (49) with pairs of primers presented in Table 2 and Table S2 in the supplemental material. Allelic exchanges were confirmed by PCR. For complementation, the *speE* gene was PCR amplified using the primers *speE*_CompF and *speE*_CompR cloned into pSC-A-amp/kan using the StrataClone PCR kit (Agilent) (see p-*speE* in Table 1).

**TABLE 2:** Primers used in this study

Primer	Sequence
*speB*_P1	TACTGGCGTGCCGTTCGATATGGCCACTTCTGGTCGTGCGGTGTAGGCTGGAGCTGCTTC
*speB*_P2	TAATAGCGCGATCGGAGGTCAGGCCGCCAATCACTGGCGTCATATGAATATCCTCCTTAG
*speB*_F	GCCTAACGACGCGGAAGG
*speB*_R	GTTTTACCCGTGCGCATCG
*speC*_F	CGCCATCTCCTTACATTCTCTC
*speC*_R	GTTGATTTTCGCTGGTTACTCC
*speE*_P1	GGCATGAAACGCTACACGACCAGTTTGGGCAGTACTTTGGTGTAGGCTGGAGCTGCTT
*speE*_P2	GATTGTAATAACGGCATTTCAGGCCAGAGGCGAGAAACATATGAATATCCTCCTTAG
*speE*_F	AGCTATTATGTTGCGCCCTT
*speE*_R	AGCCATGCAGTTTCAGTTTTT
*speE*_CompF	ATCTCGAGCCTTAAGCCTGCTTGCCTACG
*speE*_CompR	ATCTCGAGGTTTTGGCGTAGCAGATATCG
*speG*_P1	GAAGCCTTTGTTGAACTCTCTGATCTGTATGATAAGCGTGTAGGCTGGAGCTGCTTC
*speG*_P2	ATAGAGATTGAGAACGGTAAAGCCATAGTCCATTGCCATATGAATATCCTCCTTAG
*speG*_F	GCCGCGTTATTACCCCCTAA
*speG*_R	GGGTTTACACCATCAAAAATACGA

10.1128/mSphere.00414-19.2 TABLE S1 Supplemental strains used in this study. Download Table S1, DOCX file, 0.02 MB. Copyright © 2019 Chagneau et al. 2019 Chagneau et al. This content is distributed under the terms of the Creative Commons Attribution 4.0 International license.

10.1128/mSphere.00414-19.3 TABLE S2 Supplemental primers used in this study. Download Table S2, DOCX file, 0.01 MB. Copyright © 2019 Chagneau et al. 2019 Chagneau et al. This content is distributed under the terms of the Creative Commons Attribution 4.0 International license.

For the megalocytosis assay, genotoxicity quantification, DNA cross-linking assay, and *N*-myristoyl-d-asparagine quantification, E. coli strains were pregrown overnight at 37°C with shaking in Dulbecco’s modified Eagle’s medium (DMEM)-HEPES (Gibco), a polyamine-free medium. Overnight cultures were then diluted 1:50 in DMEM-HEPES and grown until they reached an optical density at 600 nm (OD_600_) of 0.6 and then processed for experiments.

For the autotoxicity assay, E. coli strains and derivatives were pregrown in LB to reach an exponential growth (OD_600_ = 0.4). A total of 2 × 10^6^ bacteria/ml were then inoculated in LB and grown for 17 h before being plated on LB agar plates to determine CFU counts.

### Determining the megalocytosis and genotoxic effect induced by colibactin.

The megalocytosis and genotoxic effect induced by colibactin were determined as previously described (35). Briefly, HeLa cells were dispensed in a 96-well cell culture plate (5 × 10^3^ to 7.5 × 10^3^ cells/well) and incubated for 24 h. For bacterial infections, cell cultures were infected with a multiplicity of infection (number of bacteria per HeLa cell at the onset of infection) ranging from 100 to 400. Four hours postinoculation, cells were washed 3 times with Hanks balanced salt solution (HBSS) and incubated in cell culture medium with 200 μg/ml gentamicin until analysis.

For megalocytosis quantification, cells were incubated for 72h before protein staining with methylene blue (1% wt/vol in 0.01 M Tris-HCl [pH 8.5]). The methylene blue was extracted with 01. N HCl. The quantification of staining was measured at OD_660_.

For H2AX phosphorylation quantification, cells were incubated for 3 h overnight before fixation with formaldehyde, permeabilization, and blocking, as previously described (35). Cells were then incubated for 2 h at room temperature with rabbit monoclonal anti-γ-H2AX antibody 9718 (1:200 [Cell Signaling Technology]). An infrared fluorescent IRDyeTM800CW-conjugated goat anti-rabbit secondary antibody (1:200 [Rockland]) was used to detect γ-H2AX. DNA was counterstained with RedDot2 (1:500 [Biotium]). DNA and γ-H2AX were visualized simultaneously using an Odyssey Infrared Imaging Scanner (Li-Cor Biosciences) with 680- and 800-nm channels. Relative fluorescence units for γ-H2AX per cell (as determined by γ-H2AX divided by DNA content) were divided by untreated controls. Results were then divided by the mean ratio obtained for the wild-type strain after infection with the same infectious dose to determine percentage change in phosphorylation of H2AX levels relative to this reference strain.

### DNA cross-linking assay.

The assay was performed as previously described (14). Briefly, linearized DNA was obtained by digesting pUC19 plasmid with BamHI (NEB). Purified linearized DNA was quantified and diluted to obtain a 200-ng/μl stock solution. For bacterium-DNA interactions, 1 × 10^6^ to 6 × 10^6^ CFU were cultivated with linearized DNA for 4 h at 37°C without shaking. Following centrifugation for 5 min at 5,000 × *g* to pellet bacteria, the DNA present in the supernatants was purified using the PCR purification kit (Qiagen) according to the manufacturer’s recommendations.

Denaturing agarose gel was prepared by dissolving 1.0 g of agarose in 100 ml of a 100 mM NaCl and 2 mM EDTA solution (pH 8.0). The gel was then soaked (2 h) in an alkaline running buffer solution (40 mM NaOH and 1 mM EDTA [pH ∼12.0]). One hundred nanograms of each DNA sample was loaded on the agarose gel. The gel was run for 45 min at 1 V/cm and then 2 h at 2 V/cm. The gel was then neutralized for a total of 45 min in a 100 mM Tris (pH 7.4) buffer solution containing 150 mM NaCl, and the neutralizing solution was refreshed every 15 min. The gel was stained with GelRed for 20 min and revealed with UV exposure using the ChemiDoc imaging system (Bio-Rad).

### *N*-Myristoyl-d-asparagine (colibactin prodrug motif) quantification by liquid chromatography-mass spectrometry.

The colibactin prodrug motif was quantified as previously described (36). Briefly, precultivated strains were grown in DMEM-HEPES at 37°C for 18 h under shaking (240 rpm). Supernatants of cultures were obtained by centrifugation of bacterial cells at 3,200 × *g* for 15 min and were filtered on 0.2-μm-pore membranes. Each strain was cultured in triplicate (derived from three independent clones), and each supernatant was analyzed by liquid chromatography-tandem mass spectrometry (LC-MS/MS).

Quantification experiments were performed with ultraperformance liquid chromatography high-resolution/heated electrospray ionization mass spectrometry (UPLC-HR/HESI-MS). The data were recorded on a Thermo Scientific Q Exactive hybrid quadrupole-Orbitrap mass spectrometer coupled to a Dionex Ultimate 3000 UPLC. The following solvent gradient (A = H_2_O + 0.1% formic acid, B = acetonitrile + 0.1% formic acid with B at 30% from0 to 1 min, 30 to 95% from 1 to 6 min, and 95% from 6 to 7 min at a flow rate of 0.5 ml/min) was used on a Phenomenex Kinetex 5-μm EVO C_18_ (50- by 2.1-mm) column at 30°C. The mass spectrometer was operated in positive-ionization mode at a scan range of 200 to 500 *m*/*z* and a resolution of 35,000. The spray voltage was set to 3.5 kV, the S-lens to 35, the auxiliary gas heater temperature to 438°C, and the capillary temperature to 270°C. Absolute quantification was achieved by using a Schotten-Baumann reaction-derived *N*-myristoyl-l-asparagine (isomer of the *N*-myristoyl-d-asparagine colibactin cleavage product) as a standard. Data were obtained from undiluted cell-free sample supernatants and analyzed for *N*-myristoyl-d-asparagine, and concentrations were calculated using Thermo Xcalibur 2.2 Quan Browser.

### Statistical analysis.

Statistical analyses were conducted using GraphPad Prism 6.01. The mean and the standard error of the mean (SEM) are shown in the figures, unless otherwise stated. *P* values were calculated by a one-way analysis of variance (ANOVA) followed by a Bonferroni *post hoc* test. A *P* value of <0.05 was considered statistically significant.
