# Supplementary figures and images for: The Polyamine Spermidine Modulates the Production of the Bacterial Genotoxin Colibactin
Source: mSphere. 2019 Oct 2;4(5):e00414-19. doi: 10.1128/mSphere.00414-19 (PMC6796968; doi:10.1128/mSphere.00414-19)

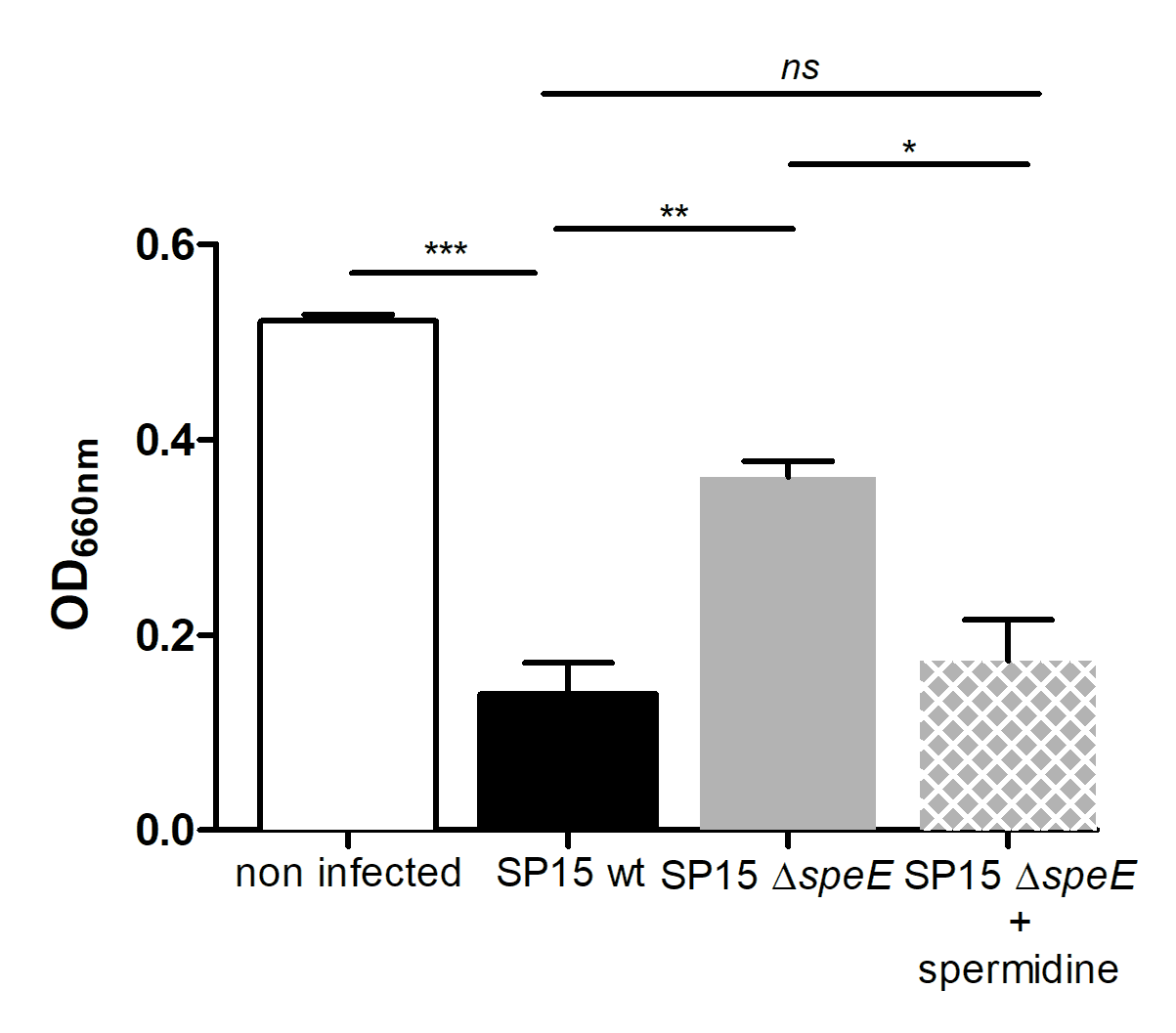

Supplement: FIG S1 [file mSphere.00414-19-sf001.tif]

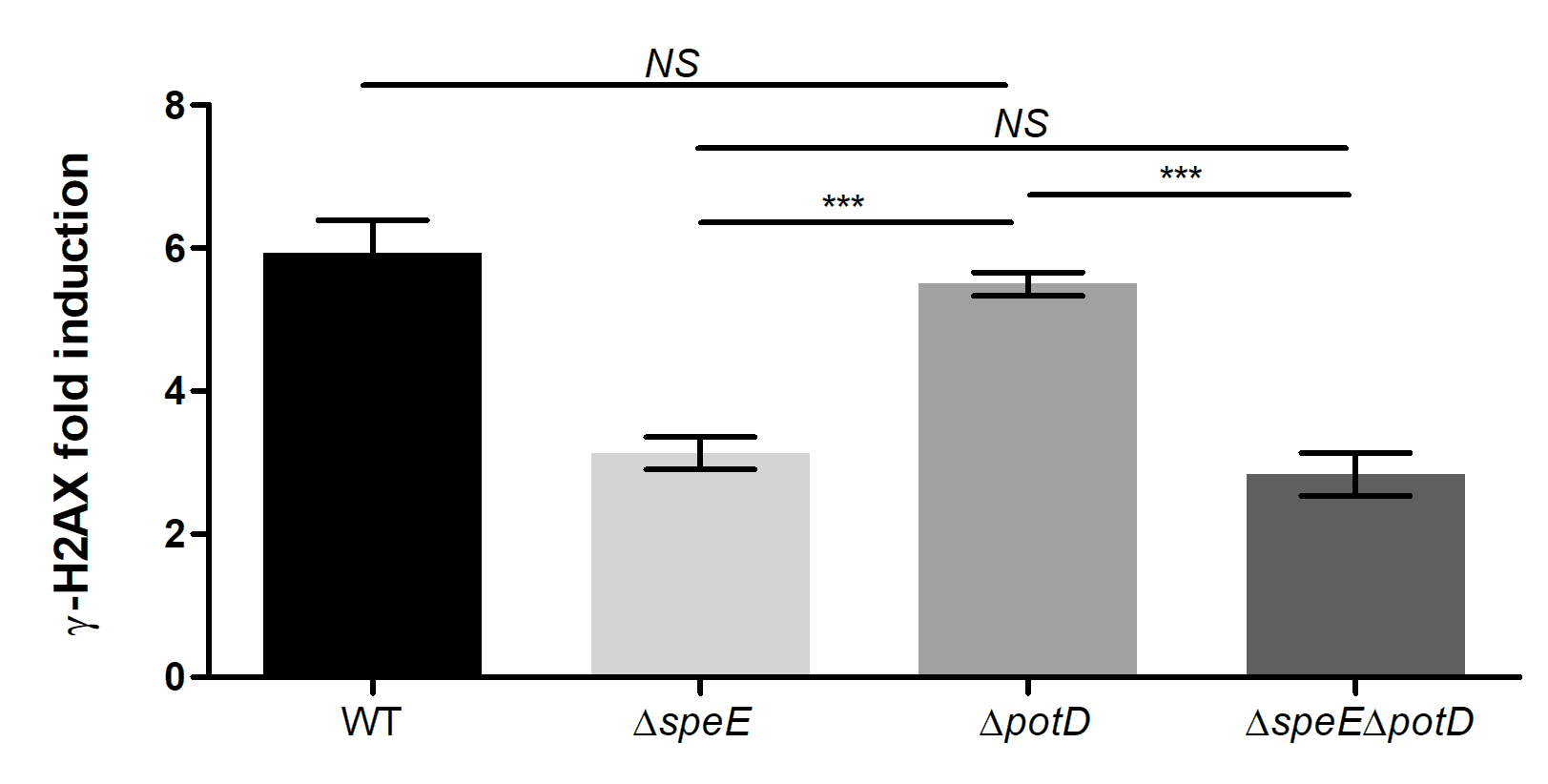

Supplement: FIG S2 [file mSphere.00414-19-sf002.tif]

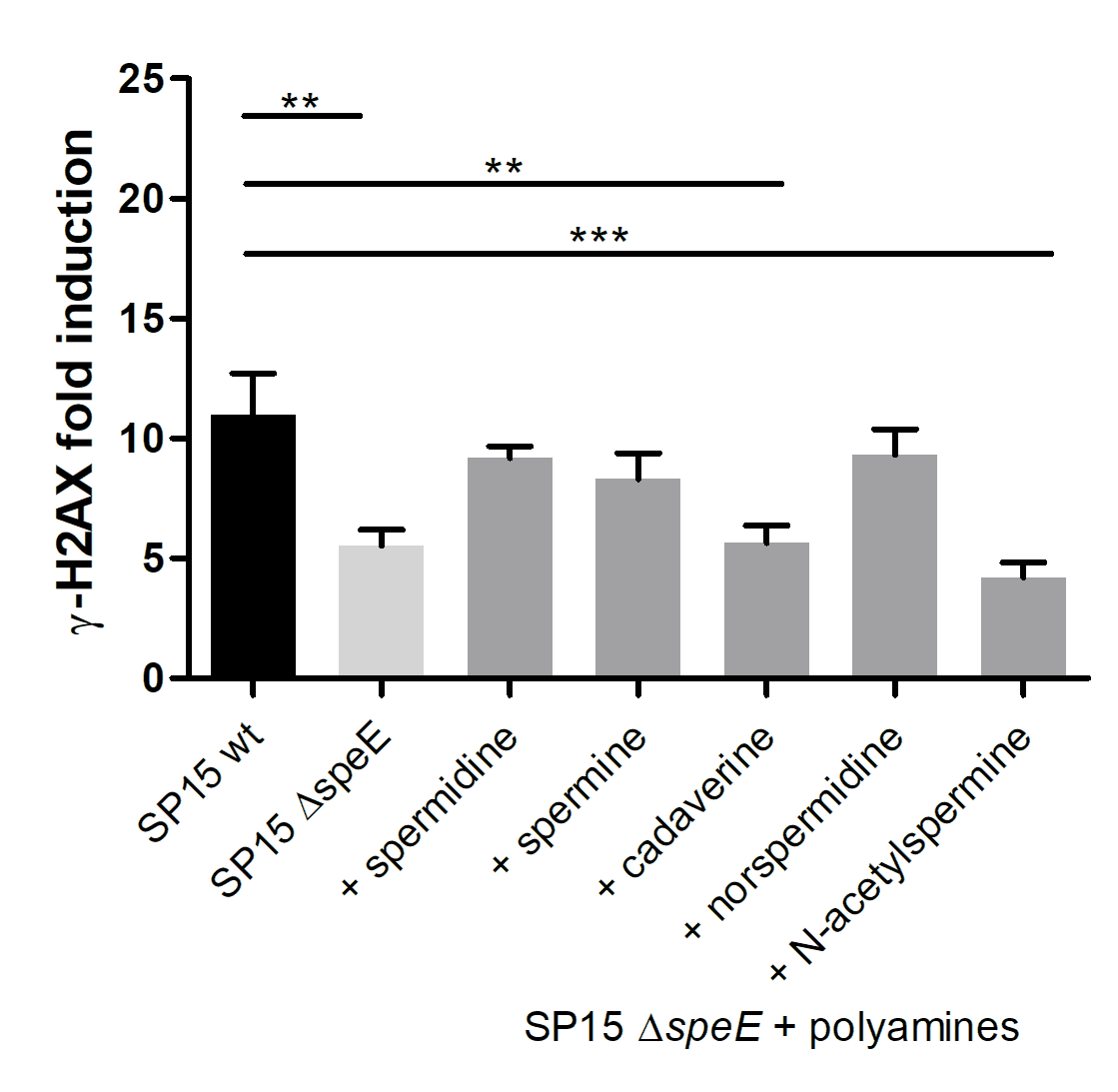

Supplement: FIG S3 [file mSphere.00414-19-sf003.tif]

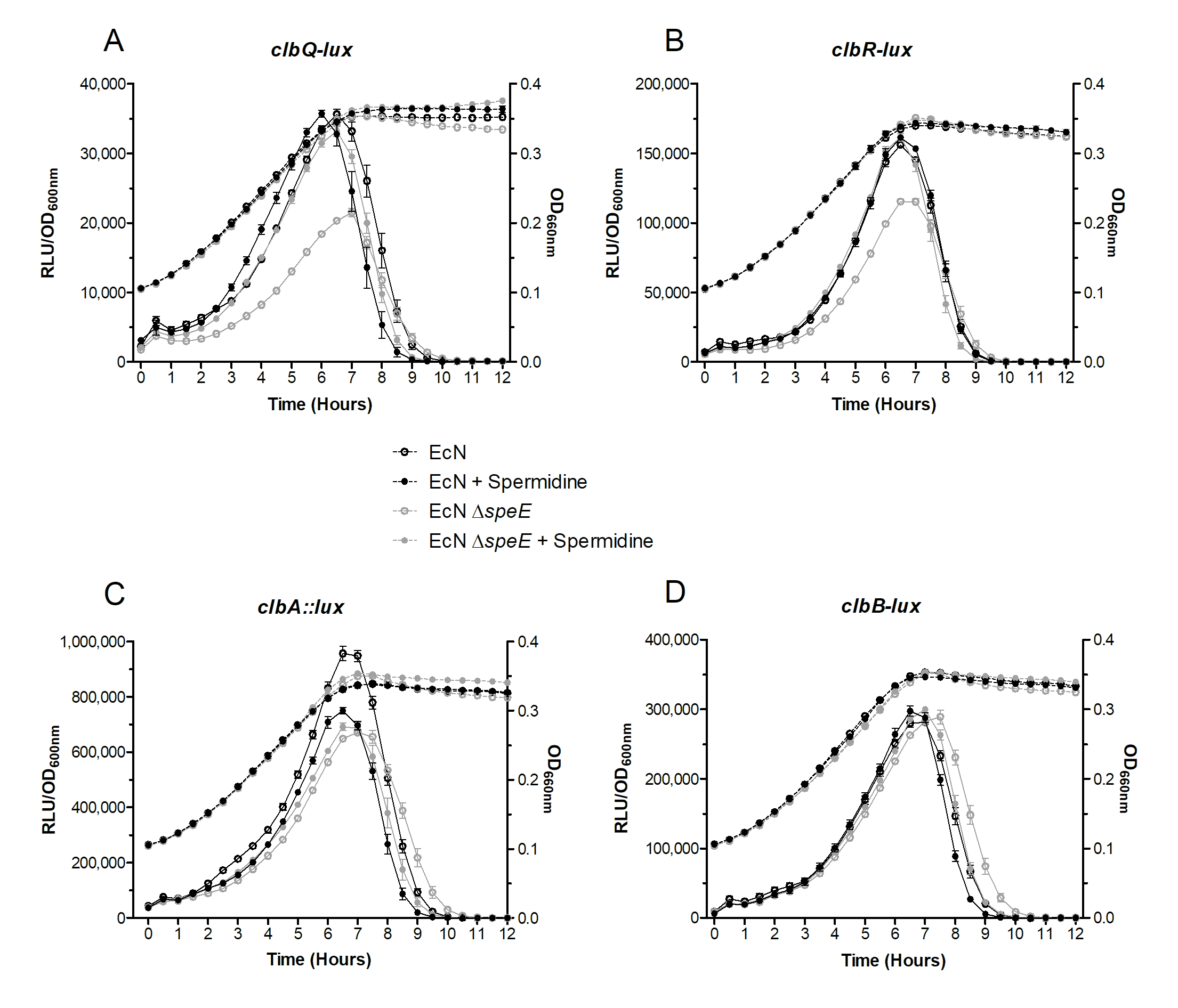

Supplement: FIG S5 [file mSphere.00414-19-sf005.tif]
