# Supplementary material for: The Polyamine Spermidine Modulates the Production of the Bacterial Genotoxin Colibactin
Source: mSphere. 2019 Oct 2;4(5):e00414-19. doi: 10.1128/mSphere.00414-19 (PMC6796968; doi:10.1128/mSphere.00414-19)
Supplement: TEXT S1 [file mSphere.00414-19-s0001.docx]

**SUPPLEMENTARY MATERIAL AND METHODS**

**Luciferase measurements**

Promoter activities of genes *clbA*, *clbB*, *clbQ*, and *clbR* were determined by time-course quantification of luciferase as previously described (1). *E. coli* strains were grown overnight in DMEM-Hepes, and subcultured into DMEM-Hepes up to OD_600nm_ = 0.6. Hundred μL were then used to inoculate black 96-well plate (Greiner Bio-one) and grown at 37°C in a luminometer (Tecan Infinite Pro microplate reader). Both OD_600nm_ and light emission (relative light units, RLU, 6000ms aperture/sample) were recorded every 30min, simultaneously.

Reference

1. Tronnet S, Garcie C, Rehm N, Dobrindt U, Oswald E, Martin P. 2016. Iron homeostasis regulates the genotoxicity of Escherichia coli that produces colibactin. Infect Immun 84:3358–3368.
