# Supplementary material for: The Polyamine Spermidine Modulates the Production of the Bacterial Genotoxin Colibactin
Source: mSphere. 2019 Oct 2;4(5):e00414-19. doi: 10.1128/mSphere.00414-19 (PMC6796968; doi:10.1128/mSphere.00414-19)
Supplement: TABLE S1 [file mSphere.00414-19-st001.docx]

| ***E. coli* strain** | **Genotype or phenotype** | **Source** |
| --- | --- | --- |
| SP15 Δ*potD* | *potD* mutant of strain SP15, Kan^r^ | This study |
| SP15 Δ*speE*Δ*potD* | *potD* mutant of strain SP15 *speE::FRT*, Kan^r^ | This study |
| EcN *clbA-lux* | Luciferase fusion of the *clbA* gene in strain *E. coli* Nissle 1917, Kan^r^, | (1) |
| EcN *clbB-lux* | Luciferase fusion of the *clbB* gene in strain *E. coli* Nissle 1917, Kan^r^ | (1) |
| EcN *clbQ-lux* | Luciferase fusion of the *clbQ* gene in strain *E. coli* Nissle 1917, Kan^r^ | (1) |
| EcN *clbR-lux* | Luciferase fusion of the *clbR* gene in strain *E. coli* Nissle 1917, Kan^r^ | (1) |
| EcN *clbA-lux* Δ*speE* | *speE* mutant of strain *E. coli* Nissle 1917 *clbA-lux,* Kan^r^ Cm^r^ | This study |
| EcN *clbB-lux* Δ*speE* | *speE* mutant of strain *E. coli* Nissle 1917 *clbB-lux,* Kan^r^ Cm^r^ | This study |
| EcN *clbQ-lux* Δ*speE* | *speE* mutant of strain *E. coli* Nissle 1917 *clbQ-lux,* Kan^r^ Cm^r^ | This study |
| EcN *clbR-lux* Δ*speE* | *speE* mutant of strain *E. coli* Nissle 1917 *clbR-lux,* Kan^r^ Cm^r^ | This study |

1. Homburg S, Oswald E, Hacker J, Dobrindt U. 2007. Expression analysis of the colibactin gene cluster coding for a novel polyketide in Escherichia coli. FEMS Microbiol Lett 275:255–262.
