# Supplementary material for: The Polyamine Spermidine Modulates the Production of the Bacterial Genotoxin Colibactin
Source: mSphere. 2019 Oct 2;4(5):e00414-19. doi: 10.1128/mSphere.00414-19 (PMC6796968; doi:10.1128/mSphere.00414-19)
Supplement: TABLE S2 [file mSphere.00414-19-st002.docx]

| **Primer** | **Sequence** |
| --- | --- |
| *potD*_P1 | ACCGAGTACGTGCCGCCAGGACTGCTTGAACAGTTCACCAAAGAAACCGGGTGTAGGCTGGAGCTGCTTC |
| *potD*_P2 | CTGATAATACTCTTCATAAATGCTGCTGGCTGCGCCAACGTCATTCTGCCCATATGAATATCCTCCTTAG |
| *potD*_F | GTACGTGCCGCCAGGACTGC |
| *potD*_R | TAAATGCTGCTGGCTGCGCC |
